# EMBEDDING DEMENTIA-FRIENDLY TRAINING IN A HEALTH CARE SYSTEM

**DOI:** 10.1093/geroni/igz038.3121

**Published:** 2019-11-08

**Authors:** Ellen C Schneider, Maureen Dale, Krista Wells, John Gotelli, Ellen Roberts, Cristine B Henage, Jan Busby-Whitehead

**Affiliations:** 1 University of North Carolina at Chapel Hill, Chapel Hill, North Carolina, United States; 2 University of North Carolina at Chapel Hill, Division of Geriatric Medicine, Chapel Hill, North Carolina, United States; 3 UNC Hospitals - Hillsborough Campus, Hillsborough, North Carolina, United States

## Abstract

Alzheimer’s disease, the most common form of dementia, is now the 6th leading cause of death in the United States, affecting one in ten people over the age of 65. With our country’s rapidly aging population, and age being the primary known risk factor for dementia, the number of people with dementia is expected to increase from 5.8 million in 2019 to 14 million in 2050. People with dementia are hospitalized more often and have prolonged stays, poorer outcomes, higher costs, and increased readmission rates. Hospital employees have expressed the desire to have specialized training to learn how to more effectively communicate with and provide better care to patients with dementia to minimize adverse outcomes and increase patient satisfaction. To better address these identified patient and hospital employee needs, the University of North Carolina’s Center for Aging and Health (UNC CAH) is disseminating hospital-specific dementia-friendly training to four hospitals within the UNC Health Care System. The training is being delivered via online modules and follow-up didactic sessions to over 4,000 clinical and non-clinical staff who interact with patients. To monitor outcomes, pre and post training data is being collected on dementia patients’ length of stay, readmission rates, and falls. The pilot project was conducted in 2019, and results of the pilot will be presented in the poster. The dementia-friendly hospital training initiative will prepare hospitals to provide better care for people with dementia, which should lead to improved health outcomes and more positive experiences for patients, caregivers, and hospital employees.

